# Triggers for the Nrf2/ARE Signaling Pathway and Its Nutritional Regulation: Potential Therapeutic Applications of Ulcerative Colitis

**DOI:** 10.3390/ijms222111411

**Published:** 2021-10-22

**Authors:** Hu Liu, Lee J. Johnston, Fenglai Wang, Xi Ma

**Affiliations:** 1 State Key Laboratory of Animal Nutrition, College of Animal Science and Technology, China Agricultural University, Beijing 100193, China; liuhu0674@cau.edu.cn (H.L.); wangfl@cau.edu.cn (F.W.); 2 Swine Nutrition and Production, West Central Research and Outreach Center, University of Minnesota, Morris, MN 56267, USA; johnstlj@umn.edu

**Keywords:** antioxidant response element, Kelch-like ECH-associated protein 1, nuclear factor E2-related factor 2, nutritional regulation, ulcerative colitis

## Abstract

Ulcerative colitis (UC), which affects millions of people worldwide, is characterized by extensive colonic injury involving mucosal and submucosal layers of the colon. Nuclear factor E2-related factor 2 (Nrf2) plays a critical role in cellular protection against oxidant-induced stress. Antioxidant response element (ARE) is the binding site recognized by Nrf2 and leads to the expression of phase II detoxifying enzymes and antioxidant proteins. The Nrf2/ARE system is a key factor for preventing and resolving tissue injury and inflammation in disease conditions such as UC. Researchers have proposed that both Keap1-dependent and Keap1-independent cascades contribute positive effects on activation of the Nrf2/ARE pathway. In this review, we summarize the present knowledge on mechanisms controlling the activation process. We will further review nutritional compounds that can modulate activation of the Nrf2/ARE pathway and may be used as potential therapeutic application of UC. These comprehensive data will help us to better understand the Nrf2/ARE signaling pathway and promote its effective application in response to common diseases induced by oxidative stress and inflammation.

## 1. Introduction

**Figure 1:**
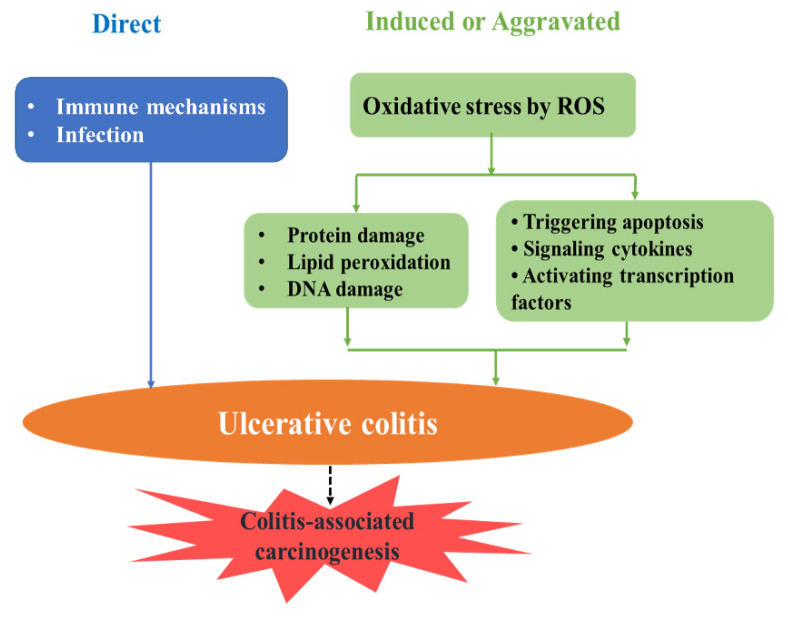
Hypothetic mechanisms involved in oxidative stress-induced ulcerative colitis, which may lead to colitis-associated carcinogenesis. Oxidative stress develops as the inflammatory cells, neutrophils, and macrophages produce large amounts of ROS, which are associated with the pathogenesis of chronic inflammatory bowel disease.

Ulcerative colitis (UC) is an idiopathic, chronic, and relapsing inflammatory bowel disease characterized by cycles of acute inflammation, ulceration, and bleeding of the colonic mucosa [1,2]. It usually has a lifelong impact with long-term disabling symptoms that increase the risk of colorectal cancer, which is the third most common malignancy in humans. Etiopathogenesis of UC remains uncertain, but many studies reported that oxidative stress is associated with the pathogenesis of chronic inflammatory bowel disease [3]. Oxidative stress develops particularly in inflammatory reactions because inflammatory cells, neutrophils, and macrophages produce large amounts of ROS (Figure 1). Accumulated ROS can cause oxidative damage to cell structures (proteins, DNA, lipids, and membranes), and they also act as chemical messengers to activate signaling pathways, such as NF-*κ*B and p38 MAPK, that affect cell proliferation, differentiation, and apoptosis [4,5]. Most current therapies for UC are effective; however, they can cause severe side effects, including diarrhea, abdominal cramps, and high blood pressure, with extended treatment periods [6]. Hence, the underlying mechanisms of UC and therapeutic strategies still require investigation for the prevention and treatment of UC (Figure 1).

Nuclear factor E2-related factor2 (Nrf2) is an important sensor protein expressed in many tissues that plays a crucial role in cellular detoxification in response to oxidative stress [6]. Upon oxidative and electrophilic insults, Nrf2 dissociates from its cytoplasmic repressor, Kelch-like ECH-associated protein 1 (Keap1), and then translocates to the nucleus [7]. As a result, expression of cytoprotective enzymes, such as NADPH: quinone oxidoreductase 1 (NQO1) and heme oxygenase 1 (HO-1), increases to enhance cellular defense against ROS, which confers protection against various deleterious oxidative stresses, inflammation, and apoptosis [8]. A growing body of evidence indicates that Nrf2 could play a potentially important role in protecting against UC through regulation of proinflammatory cytokines and induction of phase II detoxifying enzymes [9]. For this reason, Nrf2/ARE plays important roles in mitigating oxidative stress. The underlying mechanisms of controlling Nrf2/ARE activation need to be summarized and thoroughly investigated.

## 2. Triggers for Activation of the Nrf2/ARE Signaling Pathway

Nrf2 contains six highly conserved domains, known as Neh1–Neh6. The N-terminal domain of Neh2 is the major regulatory domain [10]. Neh2 attaches to Keap1 at the ETGE and DLG binding sites, which help regulate the stability of Nrf2. The Neh2 domain also contains seven lysine residues which are responsible for conjugation with ubiquitin [11]. Neh1 contains a CNC-type bZIP DNA-binding motif that allows Nrf2 to bind DNA and dimerize with other transcription factors [12]. The Neh3, Neh4, and Neh5 domains mediate transactivation of Nrf2 by interacting with coactivators, including histone acetyltransferases [13]. Recently, a seventh Neh domain (Neh7) was identified and shown to interact with the retinoic X receptor α, an Nrf2 repressor, to repress Nrf2 target gene transcription [14].

The antioxidant response element (ARE) is the consensus sequence defined as 5′-TGACnnnGC-3′, where essential nucleotides are in capitals and the ‘‘n’’ represents any nucleotide [15]. In the nucleus, the binding of Nrf2 to the ARE is facilitated via hetero dimerization with the musculoaponeurotic fibrosarcoma (Maf) protein family [16]. This binding stimulates a wide variety of downstream transcriptions that play important roles in cytoprotection and metabolism.

### 2.1. Keap1-Dependent Activation of Nrf2/ARE Pathway

Keap1 contains four characteristic domains, including a broad complex/tramtrack/bric-a-brac (BTB) domain, an intervening region (IVR), a double glycine repeat (Kelch/DGR) domain, and the C-terminal region (CTR). The BTB domain binds Cul3 and serves in the dimerization of Keap1 in cytoplasm [17]. The Kelch/DGR-CTR domain is critical for maintaining physical interaction between Keap1 and the Neh2 domain of Nrf2 [18]. The IVR contains several redox-sensitive cysteine residues which mainly modify Keap1 activity [19]. Thus, each of the four domains is thought to play a unique role in mediating Nrf2 ubiquitination and repression.

**Figure 2:**
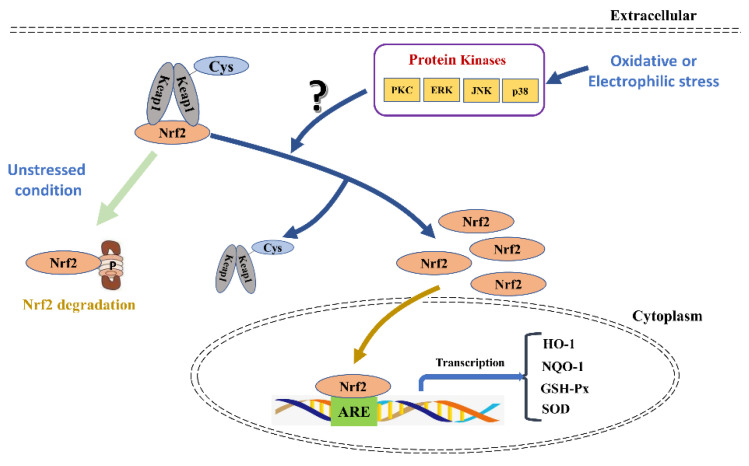
The role of Nrf2/ARE pathway in antioxidant response and its regulatory mechanism. In unstressed condition, Nrf2 synthesized in the cell is primarily bound to a complex with Keap1 and degraded by the 26S proteasomes. After oxidative or electrophilic stress, Keap1 molecules become saturated with Nrf2, and the number of newly synthesized, free Nrf2 is increased and promotes its translocation to the nucleus. In the nucleus, binding of Nrf2 to the ARE is facilitated and transcription of a wide variety of downstream are stimulated, which play an important role in cytoprotection and metabolism.

Under basal conditions, Nrf2 synthesized in the cell primarily binds to a complex with Keap1 so that concentration of free Nrf2 is low in homeostasis (Figure 2) [20]. This phenomenon could be explained by the “Hinge and latch” model, which indicates that two Keap1 bind, respectively, to the ETGE motif (hinge) and the DLG motif (latch) of Nrf2 in a favorable position which promotes Nrf2 to be ubiquitinated and degraded by 26S proteasomes [17]. After oxidative or electric stimulus, Keap1 conformation is modified to release Nrf2 from the low-affinity DLG motif [21]. However, Nrf2 remains attached to Keap1 with the high-affinity ETGE motif (hinge). Consequently, polyubiquitination and subsequent proteasomal degradation are broken [22]. The end result is that Keap1 molecules become saturated with Nrf2, and concentration of newly synthesized, free Nrf2 increases [6]. These changes activate Nrf2 and promote its translocation to the nucleus (Figure 2).

### 2.2. Keap1-Independent Activation of Nrf2/ARE Pathway

#### 2.2.1. Protein Kinase C

Protein Kinase C (PKC) is a family of phospholipid-dependent serine/threonine kinases. PKC activation increases concentration of the antioxidant proteins GST, SOD, and γ-GCS and depresses ROS content in rats, leading to an anti-oxidative effect [23]. Previous studies have also demonstrated that PKC plays important roles in the activation and expression of Nrf2. Lin et al. reported that Nrf2 and phase II detoxification gene expression could be induced via activation of the PKC signaling pathways [24]. In an in vitro model, Guo et al. asserted that protocatechualdehyde provided neuroprotection via the PKC pathway to promote Nrf2 dissociation from Keap1 and translocation into the nucleus, which up-regulated HO-1 expression [25]. Further investigation demonstrated that PKC catalyzed Nrf2 activation by phosphorylation of Nrf2 at Ser40, which is necessary for Nrf2 dissociation from Keap1 and nuclear translocation [26]. Since PKC has various isoforms including α, β, γ, δ, and ε, Chen et al. identified PKC-δ as the major PKC isoform that phosphorylated Nrf2 Ser40 [27]. In addition, PKC α and β work to activate Nrf2 at different (early or late) time points. These authors believe that each PKC isoform might trigger phosphorylation of Nrf2 by differentially inducing activation of Nrf2 [28]. The exact mechanism by which PKC isoforms regulate and activate Nrf2 is not fully understood and needs further investigation.

#### 2.2.2. AMP-Activated Protein Kinase

AMP-activated protein kinase (AMPK) is a metabolically sensitive serine/threonine protein kinase ubiquitously expressed in many different tissues [29]. It is a heterotrimeric complex consisting of a catalytic α-subunit and two regulatory subunits, β and γ [30]. AMPK was identified initially as a “fuel gauge” whose activation allows cells to enhance fuel oxidation in response to an increase in the AMP-to-ATP ratio [31]. The signaling pathway for AMPK to maintain cellular energy homeostasis is activated by phosphorylation at Thr172 in the activation loop of the catalytic α-subunits [32]. Interestingly, AMPK was characterized recently as a novel regulator and upstream signal for modulating the redox state of cells under oxidative stress. AMPK induces SOD and HO-1 expression via the Nrf2/ARE signaling pathway, which enables the cell to increase its antioxidant capacity and survival [33]. Using a chemical–biological approach, Zimmermann et al. revealed the positive influence of AMPK on Nrf2 activation and subsequent antioxidant enzyme production in response to oxidative stress [34]. Furthermore, several researchers indicated that activated AMPK/Nrf2 pathways exhibit anti-inflammatory effects on LPS-stimulated macrophages and microglia [30,35]. After a series of studies investigating the potential mechanism of AMPK/Nrf2dependent pathway, Joo et al. reported that phosphorylation of Nrf2 at the Ser550 residue by AMPK promotes Nrf2 dissociation from Keap1 and nuclear accumulation of Nrf2 for ARE-driven gene transactivation [36]. Notably, Sid et al. discovered that 5-aminoimidazole-4-carboxamide riboside could induce Nrf2 activation to modulate the redox state of human hepatocarcinoma cells via an AMPK-independent mechanism [31]. In summary, studies are in progress that aim for a complete understanding of the crosstalk between AMPK and the transcription of Nrf2.

#### 2.2.3. Mitogen-Activated Protein Kinases

Mitogen-activated protein kinases (MAPKs) belong to a family of serine/threonine kinases and play a central role in coupling various extracellular signals to a variety of biological processes, such as gene expression, cell proliferation, cell differentiation, and cell death [37]. To date, three MAPKs have been extensively studied: extracellular signal-regulated kinases (ERK), c-Jun NH2-terminal kinases (JNKs, also called stress-activated protein kinases), and p38 [38]. Activity of these MAPKs is induced through phosphorylation of their threonyl and tyrosyl residues by a dual specificity kinase termed MAP kinase kinase (MAPKK), which is phosphorylated and activated by an upstream kinase generally called MAPK kinase kinase (MAPKKK) [39]. These kinases are able to activate JNK through MKK4 and p38 through MKK3 or MKK6, but they do not affect the ERK pathway [40]. Although JNK, p38, and ERK may be regulated by different upstream kinases, they are preferentially activated by various stress stimuli and are involved in the regulation of stress signals.

##### Extracellular-Signal-Regulated Kinase 1/2

Extracellular-signal-regulated kinases 1 and 2 (ERK1/2) are isoforms of the “classical” MAPK and rely on the Thr–Glu–Tyr (TEY) activation motif. Both ERK1 and ERK2 are activated by growth factors and play an important role in regulation of cell proliferation and cell differentiation [41]. ERK1/2 is activated by MAP/ERK kinase 1 (MEK1) and MAP/ERK kinase 2 (MEK2), which are referred to as MEK1/2 [42]. Activated ERK1/2 phosphorylates many substrates, including protein kinases such as ribosomal S6 kinase (RSK), and the transcription factors Elk1 and Nrf2 [43]. ERK1/2 is an important contributor to cell proliferation and defense. Wang et al. suggested that the antioxidant pathway activated by gastrodin involves ERK1/2 phosphorylation, which increases Nrf2 nuclear translocation and leads to an elevation of phase II detoxifying enzyme and antioxidant enzyme levels [44]. Hu et al. demonstrated that up-regulating the phosphorylation of ERK 1/2 in rats could significantly activate Nrf2/ARE pathways and induce Nrf2 nuclear translocation with enhances HO-1 and NQO1 expression [43].

##### C-Jun N-Terminal Kinases and p38

C-Jun N-terminal kinases (JNKs) are also called stress-activated protein kinases (SAPKs). Recent studies have suggested that JNK and p38 play critical roles in mitigating oxidative stress. Vari et al. reported that JNK-mediated phosphorylation of Nrf2 plays an essential role in the expression of antioxidant proteins and phase II detoxifying enzymes induced by protocatechuic acid [45]. Ma et al. provided evidence that expression of Nrf2 could be induced by activation of p38 MAPK signaling in glioma cells [46]. Additionally, p38 or JNK may play a role without activation of the Nrf2/ARE pathway. Jiang et al. suggested that activation of p38/JNK but attenuation of the Nrf2/Akt pathway plays an important role in the suppression of gastric cancer by diallyl trisulfide [47]. Evidently, Nrf2 plays dual roles in cancer prevention and progression depending on the cellular context and environment. These authors suggest that a better understanding of Nrf2 is necessary to understand this balance between antioxidant pathways and the inhibition of tumor progression.

##### Phosphatidylinositol-3-Kinase/Protein Kinase B

Phosphatidylinositol-3-kinase (PI3K) is a lipid kinase that generates phosphatidylinositol-3,4,5-trisphosphate (PI (3,4,5) P3), which is a second messenger essential for translocation of Akt to the plasma membrane where it is phosphorylated [48]. Activation of Akt plays a pivotal role in fundamental cellular functions, such as cell proliferation and survival, by phosphorylating a variety of substrates [49]. Qi et al. demonstrated that activation and crosstalk between PI3K/Akt and Nrf2/HO-1 signaling pathways plays a potential role in regulating the hormesis of Z-ligustilide in PC12 cells under oxygen and glucose deprivation [50]. Likewise, Lee et al. indicated that ROS generation and/or activation of PI3K/Akt signaling regulates cell survival and Nrf2-driven HO-1 expression in sulforaphane-treated cells [51].

#### 2.2.4. Histone Modifications

##### Histone Deacetylases

Acetylation of lysine residues in histones or other transcription factors plays an important role in the regulation of transcription and gene expression [52]. Histone acetyltransferases (HATs) and histone deacetylases (HDACs) include a series of enzymes that regulate acetylation/deacetylation, which alter many physiological and pathological processes [53]. HDAC inhibition can induce hyperacetylation of target proteins, leading to an alteration of target gene transcription and expression [54]. Recent studies indicated that Nrf2 is a protein acetylation target. Nrf2 acetylation promotes its translocation capacity and downstream gene expression, thus increasing oxidative and inflammatory protection in animal and cell models [54,55]. Known as HDAC inhibitor, sodium butyrate can similarly induce Keap1/Nrf2 dissociation, Nrf2 nuclear translocation, and expression of downstream antioxidant responses [56]. Interestingly, Mercado et al. reported that reduced HDAC2 activity in COPD may account for increased Nrf2 acetylation, reduced Nrf2 stability, and impaired anti-oxidant defenses [57]. Mercado explained that Class I and II HDACs are inhibited in a COPD model, which leads to increased acetylation of Nrf2, decreasing stability and anti-oxidant potential. Similarly, inhibition of HDAC activity by TSA can cause acetylation of other residues in Nrf2 that might play a critical role in Nrf2 protein stability [57]. Currently, studies are focused on understanding the crosstalk between the HDAC and transcription of Nrf2.

##### Silent Information Regulator 2-Related Protein 1

Silent information regulator 2-related protein 1 (Sirt1) is a NAD+-dependent class III histone deacetylase that plays important roles in proliferation, cell oxidative stress, and inflammation [58]. Previous studies have demonstrated that crosstalk between Sirt1 and the Keap1/Nrf2/ARE pathway are important in the response of cells to oxidative stress [59,60]. In brief, Sirt1 can activate Nrf2 by modifying the structure of Keap1, thereby inducing nuclear translocation of Nrf2 [61]. In the nucleus, Nrf2 combines with ARE and up-regulates the expression of antioxidant proteins and phase II detoxifying enzymes to protect against oxidative stress [58]. Zhang found that Resveratrol promotes Nrf2/ARE anti-oxidative pathway through activation of Sirt1 to resist oxidative stress [62]. In addition, the deacetylation ability of Sirt1 can inhibit the transcription activity of NF-κB p65 subunits, thereby reducing the extent of inflammation [63]. Therefore, regulating Sirt1 is a potential research direction for tissue protection. Taken together, considering multiple beneficial advantages in relieving metabolic disorders, anti-oxidative responses, and inflammation, further studies are needed to investigate the regulatory mechanisms of Sirt1 in response to multiple forms of stress.

## 3. Nutritional Regulation of Nrf2/ARE Pathway

Most recently, our understanding of how nutrition plays a potential role in the prevention and/or treatment of various chronic diseases has grown tremendously [64,65]. A balanced diet based on the food components can bring health benefits. Siracusa et al. reported that cashew nuts could have beneficial action for the treatment of colitis with antioxidant and anti-inflammatory properties [66]. Antioxidant compounds may act indirectly by enhancing the endogenous cellular antioxidant defenses, such as through activation of Nrf2 (Table 1). Nutritional components may modulate the Nrf2/ARE system and may be of fundamental importance to demonstrate beneficial effects of this system in various chronic diseases such as UC [67,68]. Therefore, additional investigations are warranted into nutritional modulation of Nrf2, which may be contributory to the development of new nutritional therapies.

### 3.1. Probiotics

Probiotics are defined as “live microorganisms that, when administrated in adequate amounts, confer a health benefit on the host” [84]. One of the dietary-based strategies currently in vogue explores probiotics for the amelioration of oxidative stress-related diseases by augmentation of antioxidant defense systems operating in the human body [85]. A large body of evidence demonstrates that probiotics could have anti-oxidative effects via the Nrf2/ARE pathway. Saeedi reported that the probiotic *Lactobacillus rhamnosus* is adequately equipped with multifactorial anti-oxidative and anti-inflammatory defenses in a Nrf2-dependent system [69]. Likewise, *Lactobacillus gasseri* possesses distinctive abilities to modulate Nrf2-mediated cytoprotection and cell surface antigens to influence crosstalk between DCs and enterocytes [70]. Besides *Lactobacillus*, probiotic *Bacillus* has also shown strong anti-oxidative potential. *Bacillus amyloliquefaciens* increased gene expression of antioxidant enzymes and elevated Nrf2 concentration in jejunum. These characteristics may serve as a potential substitute for antibiotics [71].

### 3.2. Prebiotics

Prebiotics are food ingredients selectively metabolized by beneficial intestinal bacteria [86]. Dietary modulation of gut microflora by prebiotics is designed to improve health by stimulating the numbers and/or activities of *Bifidobacteria* and *Lactobacilli* [87]. Having an ‘optimal’ gut microflora can increase resistance to pathogenic bacteria, increase stimulation of the immune response, and reduce the risk of cancer [88]. *Polysaccharides* are one of the major constituents of prebiotics. Both plant *polysaccharides* and bacterial *polysaccharides* are being applied to the improvement of human health. Zhao et al. reported that dietary *Lycium barbarum polysaccharide* modified the Nrf2/ARE pathway and ameliorated insulin resistance induced by a high-fat diet via activation of PI3K/Akt signaling [72]. *Alfalfa polysaccharide* and aloe *polysaccharide* can also prevent H_2_O_2_-induced oxidative damage in MEFs by activating MAPK/Nrf2 signaling pathways and suppressing NF-κB signaling pathways [73]. In addition, Choedhury et al. indicated that low fucose-containing *bacterial polysaccharide* facilitated mitochondria-dependent, ROS-induced apoptosis of human lung epithelial carcinoma via controlled regulation of MAPK-mediated Nrf2/Keap1 homeostatic signaling [74]. Chitosan oligosaccharide (COS) also showed protective effects on oxidative damage of IPEC-1 cells [75]. However, the mechanisms of action of COS involved in the modulation of several important pathways, including the suppression of NF-κB and activation of AMP-activated protein kinase, are not known clearly.

### 3.3. Short Chain Fatty Acids

Short chain fatty acids (SCFAs), mostly generated in the colon, are produced during the fermentation of dietary fibers by gut microbiota [89]. The main SCFAs include acetate, propionate, and butyrate. These SCFAs lower lumen pH in the gut and exhibit protective effects, including maintenance of IEC integrity and immune homeostasis, and suppression of inflammation [90]. Wu et al. reported that sodium butyrate enhanced physical barrier function in the intestine of young grass carp [91]. Sodium butyrate also regulates Th17/Treg cell balance to ameliorate uveitis via the Nrf2/HO-1 pathway [76]. Additionally, sodium butyrate attenuates diabetes-induced aortic endothelial dysfunction via p300-mediated transcriptional activation of Nrf2 [92]. However, currently, the Nrf2 activation effect of other SCFAs except butyrate is not understood clearly.

### 3.4. Amino Acids

In addition to serving as the substrates for protein synthesis, amino acids in diets can act as precursors for numerous metabolic pathways involved in anti-inflammatory and antioxidant activities [93]. Specifically, Li et al. elucidated that methionine plays a critical role in inducing an endogenous antioxidant response via activation of the Nrf2/ARE pathway [77]. The optimal dietary tryptophan level could improve antioxidant status and enhance immunity in blunt snout bream [78]. Besides essential amino acids, Polat et al. unveiled that glutamine induced an increase of glucose-6-phosphate dehydrogenase via the Nrf2 pathway [79]. Dietary leucine modulated the Nrf2 antioxidant signaling pathway and immune response in juvenile blunt snout bream [80]. Indeed, more studies are needed to explore the impact of amino acids in diets on antioxidant response through Nrf2/ARE pathway.

### 3.5. Polyphenolic Compounds

Polyphenols are a group of chemical substances found in plants which are characterized by the presence of aromatic ring(s) bearing one or more hydroxyl moieties [94]. Polyphenols include resveratrol and curcumin. In addition to their antibiotic and anti-inflammatory potential, polyphenols also activate Nrf2 [95,96,97].

Curcumin, a polyphenol found in the spice turmeric, strongly induces the expression of HO-1 and its activity in different brain cells by activating heterodimers of the Nrf2/ARE pathway [81]. For this reason, curcumin protective action extends to various endocrine and metabolic diseases. Resveratrol (3,40,5-trihydroxy-trans-stilbene) is a secondary plant metabolite found in grapes, red wine, and vaccinium berries [98]. Recent research has demonstrated that resveratrol can regulate Nrf2 expression. Liu et al. observed that resveratrol protected human keratinocytes from ultraviolet A-induced oxidative stress and increased antioxidant enzyme activity [82]. They hypothesized that these actions were the result of increased Nrf2 expression and its accumulation in the nucleus. Besides resveratrol, proanthocyanidin is an important antioxidant polyphenol from grape seeds. Animal studies have manifested that proanthocyanidin was effective in improving antioxidant status and reducing inflammation in weaned pigs [83]. Similarly, polyphenols in green tea and apples showed myriad benefits owing to its potent antioxidant properties [99]. As mentioned, some polyphenols such as quercetin, kaempferol, and ellagic acid also possess protective effects on ulcerative colitis attributed to Nrf2-associated antioxidant capacity [100,101,102].

## 4. Conclusions and Perspective

Accumulating evidence supported that the Nrf2/ARE pathway plays a key role in the protective mechanism of cells through the induction of phase II detoxifying and antioxidant enzymes against exogenous and endogenous damage species. Several upstream signaling pathways, including mitogen-activated protein kinases, protein kinase C, phosphatidylinositol 3-kinase, and HDAC, are implicated in the regulation of Nrf2/ARE activity. Some signals may also work together to regulate activation of Nrf2 and translation of ARE through cooperation with other signals. Nutritional compounds as described in this review have been studied and indicated as effective modulation of the Nrf2/ARE pathway. Several classes among them have manifested their benefits for the management of colitis. However, more mechanistic studies are needed to elucidate which upstream pathways are involved in the activation processes.

Taken together, our current understanding of the molecular mechanism in activation of the Nrf2/ARE defense pathway is in its early phase. Much more researches are needed to investigate the mechanism and crosstalk of various upstream signaling pathways. Furthermore, special attention to nutritional supplements that may promote Nrf2/ARE signaling pathway is necessary. Nutritional regulation of UC with food components may reveal “nutritional therapies” in the defense against oxidative stress and inflammation via activation of the Nrf2/ARE pathway.

## Figures and Tables

**Table 1:** Modulation of Nrf2/ARE signaling pathways by nutritional components.

Nutritional Compounds	Components	Conclusions	Reference
Probiotics	*Lactobacillus rhamnosus*	Multifactorial anti-oxidative and anti-inflammatory defenses in a Nrf2-dependent system	[69]
	*Lactobacillus gasseri*	Modulation of Nrf2-mediated cytoprotection and cell surface antigens	[70]
	*Bacillus amyloliquefaciens*	Increase in gene expression of antioxidant enzymes and elevated Nrf2 concentration in jejunum	[71]
Prebiotic	*Lycium barbarum polysaccharide*	Regulation of the Nrf2/ARE pathway via activation of PI3K/Akt signaling	[72]
	*Alfalfa polysaccharide*	Prevention of H2O2-induced oxidative damage by activating MAPK/Nrf2 signaling pathways	[73]
	*Bacterial polysaccharide*	Regulation of MAPK-mediated Nrf2/Keap1 homeostatic signaling	[74]
	*Chitosan oligosaccharide*	Protective effects on oxidative damage of IPEC-1 cells	[75]
Short chain fatty acids	Butyrate	Regulation of Th17/Treg cell balance to ameliorate uveitis via the Nrf2/HO-1 pathway	[76]
Amino acids	Methionine	Inducing an endogenous antioxidant response via activation of the Nrf2-ARE pathway	[77]
	Tryptophan	Improvement of antioxidant status and enhancement of immunity in blunt snout bream	[78]
	Glutamine	Increase in glucose-6-phosphate dehydrogenase via Nrf2 pathway	[79]
	Leucine	Modulation of Nrf2 antioxidant signaling pathway and immune response	[80]
Polyphenolic	Curcumin	Activation of heterodimers of the Nrf2/ARE pathway and increase in expression of HO-1	[81]
	Resveratrol	Modulation of antioxidant enzyme activity protecting human keratinocytes from oxidative stress	[82]
	Proanthocyanidin	Effective in improving antioxidant status and reducing inflammation in weaned pigs	[83]

## Data Availability

Not applicable.

## References

[1] Jena G., Trivedi P.P., Sandala B. (2012). Oxidative stress in ulcerative colitis: An old concept but a new concern. Free Radic. Res..

[2] Babbs C.F. (1992). Oxygen radicals in ulcerative-colitis. Free Radic. Biol. Med..

[3] Roessner A., Kuester D., Malfertheiner P., Schneider-Stock R. (2008). Oxidative stress in ulcerative colitis-associated carcinogenesis. Pathol. Res. Pract..

[4] Wang Z.Q., Li S., Cao Y., Tian X.F., Zeng R., Liao D.F., Cao D.L. (2016). Oxidative stress and carbonyl lesions in ulcerative colitis and associated colorectal cancer. Oxid. Med. Cell. Longev..

[5] He L., He T., Farrar S., Ji L.B., Liu T.Y., Ma X. (2017). Antioxidants maintain cellular redox homeostasis by elimination of reactive oxygen species. Cell Physiol. Biochem..

[6] Qin S., Hou D.X. (2016). Multiple regulations of Keap1/Nrf2 system by dietary phytochemicals. Mol. Nutr. Food Res..

[7] Jaramillo M.C., Zhang D.D. (2013). The emerging role of the Nrf2-Keap1 signaling pathway in cancer. Genes Dev..

[8] Zhang Y.L., Guan L., Wang X.F., Wen T., Xing J.J., Zhao J.Y. (2008). Protection of chlorophyllin against oxidative damage by inducing HO-1 and NQO1 expression mediated by PI3K/Akt and Nrf2. Free Radic. Res..

[9] Khodir A.E., Atef H., Said E., ElKashef H.A., Salem H.A. (2017). Implication of Nrf2/HO-1 pathway in the coloprotective effect of coenzyme Q10 against experimentally induced ulcerative colitis. Inflammopharmacology.

[10] Zhang D.D., Lo S.C., Cross J.V., Templeton D.J., Hannink M. (2004). Keap1 is a redox-regulated substrate adaptor protein for a Cul3-dependent ubiquitin ligase complex. Mol. Cell Biol..

[11] McMahon M., Thomas N., Itoh K., Yamamoto M., Hayes J.D. (2006). Dimerization of substrate adaptors can facilitate cullin-mediated ubiquitylation of proteins by a “Tethering” mechanism—A two-site interaction model for the Nrf2-Keap1 complex. J. Biol. Chem..

[12] Moi P., Chan K., Asunis I., Cao A., Kan Y.W. (1994). Isolation of NFE2- related factor 2 (Nrf2), a NF-E2-like basic leucine zipper transcriptional activator that binds to the tandem NF-E2/ AP1 repeat of the b-globin locus control region. Proc. Natl. Acad. Sci. USA.

[13] Nioi P., Nguyen T., Sherratt P.J., Pickett C.B. (2005). The carboxy-terminal Neh3 domain of Nrf2 is required for transcriptional activation. Mol. Cell. Biol..

[14] Wang H.Y., Liu K.H., Geng M., Gao P., Wu X.Y., Hai Y., Li Y.X., Li Y.L., Luo L., Hayes J.D. (2013). RXR alpha inhibits the NRF2-ARE signaling pathway through a direct interaction with the Neh7 domain of NRF2. Cancer Res..

[15] Li S.J., Song Z.Y., Liu T.T., Liang J., Yuan J., Xu Z.C., Sun Z.H., Lai X.P., Xiong Q.P., Zhang D.Y. (2018). Polysaccharide from Ostrea rivularis attenuates reproductive oxidative stress damage via activating Keap1-Nrf2/ARE pathway. Carbohydr. Polym..

[16] Cui G.Z., Shan L.C., Hung M.W., Lei S.W., Choi I.L., Zhang Z.J., Yu P., Hoi P.M., Wang Y.Q., Lee S.M. (2013). A novel Danshensu derivative confers cardioprotection via PI3K/Akt and Nrf2 pathways. Int. J. Cardiol..

[17] Padmanabhan B., Tong K.I., Ohta T., Nakamura Y., Scharlock M., Ohtsuji M., Kang M.I., Kobayashi A., Yokoyama S., Yamamoto M. (2006). Structural basis for defects of Keap1 activity provoked by its point mutations in lung cancer. Mol. Cell.

[18] Zhang D.D., Hannink M. (2003). Distinct cysteine residues in Keap1 are required for Keap1-dependent ubiquitination of Nrf2 and for stabilization of Nrf2 by chemopreventive agents and oxidative stress. Mol. Cell. Biol..

[19] Kobayashi M., Yamamoto M. (2006). Nrf2-Keap1 regulation of cellular defense mechanisms against electrophiles and reactive oxygen species. Adv. Enzym. Regul..

[20] Huang K.P., Gao X., Wei W.T. (2017). The crosstalk between Sirt1 and Keap1/Nrf2/ARE anti-oxidative pathway forms a positive feedback loop to inhibit FN and TGF-beta 1 expressions in rat glomerular mesangial cells. Exp. Cell Res..

[21] Cheng D., Wu R., Guo Y., Kong A.-N.T. (2016). Regulation of Keap1-Nrf2 signaling: The role of epigenetics. Curr. Opin. Toxicol..

[22] Martinez V.D., Vucic E.A., Pikor L.A., Thu K.L., Hubaux R., Lam W.L. (2013). Frequent concerted genetic mechanisms disrupt multiple components of the NRF2 inhibitor KEAP1/CUL3/RBX1 E3-ubiquitin ligase complex in thyroid cancer. Mol. Cancer.

[23] Buelna-Chontal M., Guevara-Chavez J.G., Silva-Palacios A., Medina-Campos O.N., Pedraza-Chaverri J., Zazueta C. (2014). Nrf2-regulated antioxidant response is activated by protein kinase C in postconditioned rat hearts. Free Radic. Biol. Med..

[24] Lin M.S., Zhai X.H., Wang G.Z., Tian X.F., Gao D.Y., Shi L., Wu H., Fan Q., Peng J.Y., Liu K.X. (2015). Salvianolic acid B protects against acetaminophen hepatotoxicity by inducing Nrf2 and phase II detoxification gene expression via activation of the PI3K and PKC signaling pathways. J. Pharmacol. Sci..

[25] Guo C., Wang S.Q., Duan J.L., Jia N., Zhu Y.R., Ding Y., Guan Y., Wei G., Yin Y., Xi M.M. (2017). Protocatechualdehyde protects against cerebral ischemia-reperfusion-induced oxidative injury via protein kinase C epsilon/Nrf2/HO-1 pathway. Mol. Neurobiol..

[26] Huang H.C., Nguyen T., Pickett C.B. (2002). Phosphorylation of Nrf2 at Ser-40 by protein kinase C regulates antioxidant response element-mediated transcription. J. Biol. Chem..

[27] Chen L.L., Ran Q., Xiang Y., Xiang L.X., Chen L., Li F.J., Wu J., Wu C., Li Z.J. (2017). Co-activation of PKC-delta by CRIF1 modulates oxidative stress in bone marrow multipotent mesenchymal stromal cells after irradiation by phosphorylating NRF2 Ser40. Theranostics.

[28] Lee K.M., Kang K., Lee S.B., Nho C.W. (2013). Nuclear factor-E2 (Nrf2) is regulated through the differential activation of ERK1/2 and PKC alpha/beta II by Gymnasterkoreayne B. Cancer Lett..

[29] Park J.S., Lee Y.Y., Kim J., Seo H., Kim H.S. (2016). beta-Lapachone increases phase II antioxidant enzyme expression via NQO1-AMPK/PI3K-Nrf2/ARE signaling in rat primary astrocytes. Free Radic. Biol. Med..

[30] Park S.Y., Jin M.L., Ko M.J., Park G., Choi Y.W. (2016). Anti-neuroinflammatory effect of emodin in LPS-Stimulated microglia: Involvement of AMPK/Nrf2 activation. Neurochem. Res..

[31] Sid B., Glorieux C., Valenzuela M., Rommelaere G., Najimi M., Dejeans N., Renard P., Verrax J., Calderon P.B. (2014). AICAR induces Nrf2 activation by an AMPK-independent mechanism in hepatocarcinoma cells. Biochem. Pharmacol..

[32] Hurley R.L., Anderson K.A., Franzone J.M., Kemp B.E., Means A.R., Witters L.A. (2005). The Ca^2+^/calmodulin-dependent protein kinase kinases are AMP-activated protein kinase kinases. J. Biol. Chem..

[33] Liu X.M., Peyton K.J., Shebib A.R., Wang H., Korthuis R.J., Durante W. (2011). Activation of AMPK stimulates heme oxygenase-1 gene expression and human endothelial cell survival. Am. J. Physiol. Heart Circ. Physiol..

[34] Zimmermann K., Baldinger J., Mayerhofer B., Atanasov A.G., Dirsch V.M., Heiss E.H. (2015). Activated AMPK boosts the Nrf2/HO-1 signaling axis—A role for the unfolded protein response. Free Radic. Biol. Med..

[35] Ci X.X., Zhou J.F., Lv H.M., Yu Q.L., Peng L.P., Hua S.C. (2017). Betulin exhibits anti-inflammatory activity in LPS-stimulated macrophages and endotoxin-shocked mice through an AMPK/AKT/Nrf2-dependent mechanism. Cell Death Dis..

[36] Joo M.S., Kim W.D., Lee K.Y., Kim J.H., Koo J.H., Kim S.G. (2016). AMPK facilitates nuclear accumulation of Nrf2 by phosphorylating at Serine 550. Mol. Cell. Biol..

[37] Wang J.F., Zhang L., Zhang Y., Luo M.L., Wu Q.O., Yu L.J., Chu H.Y. (2015). Transcriptional upregulation centra of HO-1 by EGB via the MAPKs/Nrf2 pathway in mouse C2C12 myoblasts. Toxicol. In Vitro.

[38] Yu R., Chen C., Mo Y.Y., Hebbar V., Owuor E.D., Tann T.H., Kong A.N.T. (2000). Activation of mitogen-activated protein kinase pathways induces antioxidant response element-mediated gene expression via a Nrf2-dependent mechanism. J. Biol. Chem..

[39] Kumar H., Kim I.S., More S.V., Kim B.W., Choi D.K. (2014). Natural product-derived pharmacological modulators of Nrf2/ARE pathway for chronic diseases. Nat. Prod. Rep..

[40] Ichimura Y., Waguri S., Sou Y., Kageyama S., Hasegawa J., Ishimura R., Saito T., Yang Y.J., Kouno T., Fukutomi T. (2013). Phosphorylation of p62 activates the Keap1-Nrf2 pathway during selective autophagy. Mol. Cell.

[41] Mao L.M., Tang Q.S., Samdani S., Liu Z.G., Wang J.Q. (2004). Regulation of MAPK/ERK phosphorylation via ionotropic glutamate receptors in cultured rat striatal neurons. Eur. J. Neurosci..

[42] Nishimoto S., Nishida E. (2006). MAPK signalling: ERK5 versus ERK1/2. EMBO Rep..

[43] Hu Y.Y., Duan M.Y., Liang S., Wang Y., Feng Y. (2015). Senkyunolide I protects rat brain against focal cerebral ischemia-reperfusion injury by up-regulating p-Erk1/2, Nrf2/HO-1 and inhibiting caspase 3. Brain Res..

[44] Wang X.L., Xing G.H., Hong B., Li X.M., Zou Y., Zhang X.J., Dong M.X. (2014). Gastrodin prevents motor deficits and oxidative stress in the MPTP mouse model of Parkinson’s disease: Involvement of ERK1/2-Nrf2 signaling pathway. Life Sci..

[45] Vari R., D’Archivio M., Filesi C., Carotenuto S., Scazzocchio B., Santangelo C., Giovannini C., Masella R. (2011). Protocatechuic acid induces antioxidant/detoxifying enzyme expression through JNK-mediated Nrf2 activation in murine macrophages. J. Nutr. Biochem..

[46] Ma L.N., Liu J., Zhang X.L., Qi J.Q., Yu W.G., Gu Y.C. (2015). p38 MAPK-dependent Nrf2 induction enhances the resistance of glioma cells against TMZ. Med. Oncol..

[47] Jiang X.Y., Zhu X.S., Xu H.Y., Zhao Z.X., Li S.Y., Li S.Z., Cai J.H., Cao J.M. (2017). Diallyl trisulfide suppresses tumor growth through the attenuation of Nrf2/Akt and activation of p38/JNK and potentiates cisplatin efficacy in gastric cancer treatment. Acta Pharmacol. Sin..

[48] Osaki M., Oshimura M., Ito H. (2004). PI3K-Akt pathway: Its functions and alterations in human cancer. Apoptosis.

[49] Hamdulay S.S., Wang B.F., Birdsey G.M., Ali F., Dumont O., Evans P.C., Haskard D.O., Wheeler-Jones C.P., Mason J.C. (2010). Celecoxib activates PI-3K/Akt and mitochondrial redox signaling to enhance heme oxygenase-1-mediated anti-inflammatory activity in vascular endothelium. Free Radic. Biol. Med..

[50] Qi H.Y., Han Y.F., Rong J.H. (2012). Potential roles of PI3K/Akt and Nrf2-Keap1 pathways in regulating hormesis of Z-ligustilide in PC12 cells against oxygen and glucose deprivation. Neuropharmacology.

[51] Lee Y.J., Jeong H.Y., Kim Y.B., Lee Y.J., Won S.Y., Shim J.H., Cho M.K., Nam H.S., Lee S.H. (2012). Reactive oxygen species and PI3K/Akt signaling play key roles in the induction of Nrf2-driven heme oxygenase-1 expression in sulforaphane-treated human mesothelioma MSTO-211H cells. Food Chem. Toxicol..

[52] McMahon M., Campbell K.H., MacLeod A.K., McLaughlin L.A., Henderson C.J., Wolf C.R. (2014). HDAC inhibitors increase NRF2-Signaling in tumour cells and blunt the efficacy of co-adminstered cytotoxic agents. PLoS ONE.

[53] Correa F., Mallard C., Nilsson M., Sandberg M. (2011). Activated microglia decrease histone acetylation and Nrf2-inducible anti-oxidant defence in astrocytes: Restoring effects of inhibitors of HDACs, p38 MAPK and GSK3β. Neurobiol. Dis..

[54] Cai D.W., Yin S.S., Yang J., Jiang Q., Cao W.S. (2015). Histone deacetylase inhibition activates Nrf2 and protects against osteoarthritis. Arthritis Res. Ther..

[55] Chen Z.W., Ye X.Y., Tang N.W., Shen S.P., Li Z.M., Niu X.M., Lu S., Xu L. (2014). The histone acetylranseferase hMOF acetylates Nrf2 and regulates anti-drug responses in human non-small cell lung cancer. Br. J. Pharmacol..

[56] Dong W.P., Jia Y., Liu X.X., Zhang H., Li T., Huang W.L., Chen X.D., Wang F.C., Sun W.X., Wu H. (2017). Sodium butyrate activates NRF2 to ameliorate diabetic nephropathy possibly via inhibition of HDAC. J. Endocrinol..

[57] Mercado N., Thimmulappa R., Thomas C.M.R., Fenwick P.S., Chana K.K., Donnelly L.E., Biswal S., Ito K., Barnes P.J. (2011). Decreased histone deacetylase 2 impairs Nrf2 activation by oxidative stress. Biochem. Biophys. Res. Commun..

[58] Ding Y.W., Zhao G.J., Li X.L., Hong G.L., Li M.F., Qiu Q.M., Wu B., Lu Z.Q. (2016). SIRT1 exerts protective effects against paraquat-induced injury in mouse type II alveolar epithelial cells by deacetylating NRF2 in vitro. Int. J. Mol. Med..

[59] Huang K.P., Chen C., Hao J., Huang J.Y., Wang S.G., Liu P.Q., Huang H.Q. (2015). Polydatin promotes Nrf2-ARE anti-oxidative pathway through activating Sirt1 to resist AGEs-induced upregulation of fibronetin and transforming growth factor-beta 1 in rat glomerular messangial cells. Mol. Cell Endocrinol..

[60] Chen Y.T., Lin Y.C., Lin J.S., Yang N.S., Chen M.J. (2018). Sugary kefir strain *Lactobacillus mali* APS1 ameliorated hepatic steatosis by regulation of SIRT-1/Nrf-2 and gut microbiota in rats. Mol. Nutr. Food Res..

[61] Chai D.D., Zhang L., Xi S.W., Cheng Y.Y., Jiang H., Hu R. (2018). Nrf2 activation induced by Sirt1 ameliorates acute lung injury after intestinal ischemia/reperfusion through NOX4-mediated gene regulation. Cell. Physiol. Biochem..

[62] Zhang P., Li Y., Du Y., Li G., Wang L., Zhou F. (2016). Resveratrol ameliorated vascular calcification by regulating Sirt-1 and Nrf2. Transplant. Proc..

[63] Sun X., Wang P., Yao L.P., Wang W., Gao Y.M., Zhang J., Fu Y.J. (2018). Paeonol alleviated acute alcohol-induced liver injury via SIRT1/Nrf2/NF-kappa B signaling pathway. Environ. Toxicol. Pharmacol..

[64] Huang C.Y., Fan Z.J., Han D.D., Johnston L.J., Ma X., Wang F.L. (2021). Pyrroloquinoline quinone regulates the redox status in vitro and in vivo of weaned pigs via the Nrf2/HO-1 pathway. J. Anim. Sci. Biotechnol..

[65] Kang H.J., Hong Y.B., Kim H.J., Wang A.T., Bae I. (2012). Bioactive food components prevent carcinogenic stress via Nrf2 activation in BRCA1 deficient breast epithelial cells. Toxicol. Lett..

[66] Siracusa R., Fusco R., Peritore A.F., Cordaro M., D’Amico R., Genovese T., Gugliandolo E., Crupi R., Smeriglio A., Mandalari G. (2020). The antioxidant and anti-inflammatory properties of *Anacardium Occidentale* L. cashew nuts in a mouse model of colitis. Nutrients.

[67] Soyalan B., Minn J., Schmitz H.J., Schrenk D., Will F., Dietrich H., Baum M., Eisenbrand G., Janzowski C. (2011). Apple juice intervention modulates expression of ARE-dependent genes in rat colon and liver. Eur. J. Nutr..

[68] Rodriguez-Ramiro I., Ramos S., Bravo L., Goya L., Martin M.A. (2012). Procyanidin B2 induces Nrf2 translocation and glutathione S-transferase P1 expression via ERKs and p38-MAPK pathways and protect human colonic cells against oxidative stress. Eur. J. Nutr..

[69] Saeedi B.J., Robinson B.S., Owens J., Liu K., Eboka R., Darby T., Luo L., Jones D., Jones R., Neish A. (2019). Regulation of the hepatic antioxidant response by the probiotic *Lactobacillus rhamnosus* GG. FASEB J..

[70] Luongo D., Miyamoto J., Bergamo P., Nazzaro F., Baruzzi F., Sashihara T., Tanabe S., Rossi M. (2013). Differential modulation of innate immunity in vitro by probiotic strains of *Lactobacillus gasseri*. BMC Microbiol..

[71] Wang Y., Wu Y.P., Wang B.K., Cao X.F., Fu A.K., Li Y.L., Li W.F. (2017). Effects of probiotic Bacillus as a substitute for antibiotics on antioxidant capacity and intestinal autophagy of piglets. AMB Express.

[72] Yang Y., Li W., Li Y., Wang Q., Gao L., Zhao J.J. (2014). Dietary lycium barbarum polysaccharide induces Nrf2/ARE pathway and ameliorates insulin resistance induced by high-fat via activation of PI3K/AKT signaling. Oxid. Med. Cell. Longev..

[73] Wang L.X., Xie Y.H., Yang W.R., Yang Z.B., Jiang S.Z., Zhang C.Y., Zhang G.G. (2019). Alfalfa polysaccharide prevents H2O2-induced oxidative damage in MEFs by activating MAPK/Nrf(2) signaling pathways and suppressing NF-kappa B signaling pathways. Sci. Rep..

[74] Chowdhury S.R., Sengupta S., Biswas S., Sen R., Sinha T.K., Basak R.K., Adhikari B., Bhattacharyya A. (2015). Low fucose containing bacterial polysaccharide facilitate mitochondria-dependent ROS-induced apoptosis of human lung epithelial carcinoma via controlled regulation of MAPKs-mediated Nrf2/Keap1 homeostasis signaling. Mol. Carcinog..

[75] Muanprasat C., Chatsudthipong V. (2017). Chitosan oligosaccharide: Biological activities and potential therapeutic applications. Pharmacol. Ther..

[76] Chen X.Q., Su W.R., Wan T.S., Yu J.F., Zhu W.J., Tang F., Liu G.M., Olsen N., Liang D., Zheng S.G. (2017). Sodium butyrate regulates Th17/Treg cell balance to ameliorate uveitis via the Nrf2/F10-1 pathway. Biochem. Pharmacol..

[77] Wang Z.X., Liang M.C., Li H., Cai L., He H.J., Wu Q., Yang L. (2019). l-Methionine activates Nrf2-ARE pathway to induce endogenous antioxidant activity for depressing ROS-derived oxidative stress in growing rats. J. Sci. Food Agric..

[78] Ji K., Liang H.L., Ren M.C., Ge X.P., Li B., Xi B.W., Pan L.K., Yu H. (2019). Effects of dietary tryptophan levels on antioxidant status and immunity for juvenile blunt snout bream (Megalobrama amblycephala) involved in Nrf2 and TOR signaling pathway. Fish Shellfish Immunol..

[79] Polat I.H., Tarrado-Castellarnau M., Benito A., Hernandez-Carro C., Centelles J., Marin S., Cascante M. (2021). Glutamine modulates expression and function of glucose 6-phosphate dehydrogenase via Nrf2 in colon cancer cells. Antioxidants.

[80] Liang H.L., Mokrani A., Ji K., Ge X.P., Ren M.C., Xie J., Liu B., Xi B.W., Zhou Q.L. (2018). Dietary leucine modulates growth performance, Nrf2 antioxidant signaling pathway and immune response of juvenile blunt snout bream (Megalobrama amblycephala). Fish Shellfish Immunol..

[81] He H., Wang G., Gao Y., Ling W., Yu Z., Jin T. (2012). Curcumin attenuates Nrf2 signaling defect, oxidative stress in muscle and glucose intolerance in high fat diet-fed mice. World J. Diabetes.

[82] Liu Y., Chan F.X., Sun H.M., Yan J.H., Fan D.Y., Zhao D.Z., An J., Zhou D.S. (2011). Resveratrol protects human keratinocytes HaCaT cells from UVA-induced oxidative stress damage by downregulating Keap1 expression. Eur. J. Pharmacol..

[83] Wu Y., Ma N., Song P.X., He T., Levesque C., Bai Y.Y., Zhang A.Z., Ma X. (2019). Grape seed Proanthocyanidin affects lipid metabolism via changing gut microflora and enhancing propionate production in weaned pigs. J. Nutr..

[84] Fukumoto S., Toshimitsu T., Matsuoka S., Maruyama A., Oh-oka K., Takamura T., Nakamura Y., Ishimaru K., Fujii-Kuriyama Y., Ikegami S. (2014). Identification of a probiotic bacteria-derived activator of the aryl hydrocarbon receptor that inhibits colitis. Immunol. Cell Biol..

[85] Ma N., Guo P.T., Zhang J., He T., Kim S.W., Zhang G.L., Ma X. (2018). Nutrients mediate intestinal bacteria—Mucosal immune crosstalk. Front. Immunol..

[86] Yu Y.B., Wang C.H., Wang A.M., Yang W.P., Lv F., Liu F., Liu B., Sun C.X. (2018). Effects of various feeding patterns of Bacillus coagulans on growth performance, antioxidant response and Nrf2-Keap1 signaling pathway in juvenile gibel carp (Carassius auratus gibelio). Fish Shellfish Immunol..

[87] Pistol G.C., Marin D.E., Dragomir C., Taranu I. (2019). Synbiotic combination of prebiotic grape pomace extract and probiotic *Lactobacillus* sp. reduced important intestinal inflammatory markers and in-depth signalling mediators in lipopolysaccharide-treated Caco-2 cells. Br. J. Nutr..

[88] Zhao J.B., Liu P., Wu Y., Guo P.T., Liu L., Ma N., Levesque C., Chen Y.Q., Zhao J.S., Zhang J. (2018). Dietary fiber increases butyrate-producing bacteria and improves the growth performance of weaned piglets. J. Agric. Food Chem..

[89] Zhang S.M., Zhao J.W., Xie F., He H.X., Johnston L.J., Dai X.F., Wu C.D., Ma X. (2021). Dietary fiber-derived short-chain fatty acids: A potential therapeutic target to alleviate obesity-related nonalcoholic fatty liver disease. Obes. Rev..

[90] Liu H., Wang J., He T., Becker S., Zhang G., Li D., Ma X. (2018). Butyrate: A double-edged sword for health?. Adv. Nutr..

[91] Wu P., Tian L., Zhou X.Q., Jiang W.D., Liu Y., Jiang J., Xie F., Kuang S.Y., Tang L., Tang W.N. (2018). Sodium butyrate enhanced physical barrier function referring to Nrf2, JNK and MLCK signaling pathways in the intestine of young grass carp (*Ctenopharyngodon idella*). Fish Shellfish Immunol..

[92] Wu J.D., Jiang Z.P., Zhang H.N., Liang W.Z., Huang W.L., Zhang H., Li Y., Wang Z.H., Wang J.N., Jia Y. (2018). Sodium butyrate attenuates diabetes-induced aortic endothelial dysfunction via P300-mediated transcriptional activation of Nrf2. Free Radic. Biol. Med..

[93] Ma N., Ma X. (2019). Dietary amino acids and the gut-microbiome-immune axis: Physiological metabolism and therapeutic prospects. Compr. Rev. Food Sci. Food Saf..

[94] Carrato B., Sanzini E. (2005). Biologically-active phytochemicals in vegetable food. Ann. Ist. Super. Sanita.

[95] Rahman I., Biswas S.K., Kirkham P.A. (2006). Regulation of inflammation and redox signaling by dietary polyphenols. Biochem. Pharmacol..

[96] Chen Y.J., Wang J.S., Chow S.E. (2007). Resveratrol protects vascular endothelial cell from ox-LDL-induced reduction in antithrombogenic activity. Chin. J. Physiol..

[97] Bhatt S.R., Lokhandwala M.F., Banday A.A. (2011). Resveratrol prevents endothelial nitric oxide synthase uncoupling and attenuates development of hypertension in spontaneously hypertensive rats. Eur. J. Pharmacol..

[98] Rimando A.M., Kalt W., Magee J.B., Dewey J., Ballington J.R. (2004). Resveratrol, pterostilbene, and piceatannol in Vaccinium berries. J. Agric. Food Chem..

[99] Baliga M.S., Saxena A., Kaur K., Kalekhan F., Chacko A., Venkatesh P., Fayad R. (2014). Polyphenols in the prevention of ulcerative colitis: Past, present and future. Polyphen. Hum. Health Dis..

[100] Sharma A., Parikh M., Shah H., Gandhi T. (2020). Modulation of Nrf2 by quercetin in doxorubicin-treated rats. Heliyon.

[101] Rajendran P., Ammar R.B., Al-Saeedi F.J., Mohamed M.E., ElNaggar M.A., Al-Ramadan S.Y., Bekhet G.M., Soliman A.M. (2021). Kaempferol inhibits Zearalenone-induced oxidative stress and apoptosis via the PI3K/Akt-mediated Nrf2 signaling pathway: In vitro and in vivo studies. Int. J. Mol. Sci..

[102] Wei Y.Z., Zhu G.F., Zheng C.Q., Li J.J., Sheng S., Li D.D., Wang G.Q., Zhang F. (2020). Ellagic acid protects dopamine neurons from rotenone-induced neurotoxicity via activation of Nrf2 signalling. J. Cell Mol. Med..

